# Molecular Classification Guides Fertility-Sparing Treatment for Endometrial Cancer and Atypical Hyperplasia Patients

**DOI:** 10.3390/curroncol32060317

**Published:** 2025-05-30

**Authors:** Yiqin Wang, Linlin Bo, Xiaowei Fan, Nan Kang, Xiaobo Zhang, Li Tian, Rong Zhou, Jianliu Wang

**Affiliations:** 1 Department of Obstetrics and Gynecology, Peking University People’s Hospital, Beijing 100044, China; emily_wang92@163.com (Y.W.); bo_lin_lin@163.com (L.B.); tianli916916@126.com (L.T.); zhouron99@126.com (R.Z.); 2 Department of Gynecology, Fei County People’s Hospital, Jining 273400, China; 18764972797@163.com; 3 Department of Pathology, Peking University People’s Hospital, Beijing 100044, China; kn2009@163.com (N.K.); zhangxiaobo0326@163.com (X.Z.)

**Keywords:** endometrial neoplasms, molecular classification, fertility-sparing, mismatch repair deficiency, *POLE* mutation

## Abstract

Objectives: The objective of this study was to investigate the significance of molecular classification in guiding treatment decisions for patients with endometrial cancer (EC) or atypical hyperplasia (AH) undergoing fertility-sparing treatment (FST), particularly for those with non-NSMP subtypes. Methods: We conducted a retrospective cohort study involving EC/AH patients undergoing FST and molecular classification using next-generation sequencing at Peking University People’s Hospital between June 2020 and September 2023. Results: A total of 118 EC/AH patients were included, including 92 cases with NSMP, 11 with MMRd, 11 with *POLE*mut, and 4 with p53abn. (1) Of the 11 patients with MMRd, 6 achieved a complete response (CR) with 1 case receiving progestin, 3 cases showed insensitivity to the initial progestin before transitioning to a combined regimen of progestin and a PD-1 inhibitor, and 2 cases initially received progestin plus a PD-1 inhibitor. There were no significant differences in the cumulative CR rates between the MMRd and NSMP subgroups but a trend of a lower relapse-free-survival (RFS) rate for the MMRd subgroup (*p* = 0.074). (2) Of the 11 cases with *POLE*mut, 10 achieved CR but 4 relapsed. There was also a trend for a lower RFS rate in the *POLE*mut patients (*p* = 0.069) compared with the NSMP subgroup. (3) Three of the four patients with p53mut achieved CR after treatment with the GnRHa plus LNG-IUS regimen. Conclusion: The selection of appropriate regimens may improve FST outcomes in EC/AH patients with molecular classification of non-NSMP subtypes. Immunotherapy is an effective fertility-preserving approach for patients with MMRd.

## 1. Introduction

Endometrial cancer (EC) is the leading gynecologic malignancy worldwide. The prevalence of EC in women ≤40 years of age has been reported to be between 4 and 14% [1,2]. In selected young women of reproductive age with early-stage EC and atypical hyperplasia (AH), fertility-sparing treatment (FST) provides a conservative option with a similar prognosis to that of standard surgery management [3]. Progestin-based FST has been proven to have a high remission rate of 72–76% for early-stage EC [4].

First proposed in 2013, the Cancer Genome Atlas (TCGA) differentiates EC into four prognostically significant groups and was later recommended by the 5th WHO classification [5,6]. Molecular classification provides important information for prognosis and personalized treatment options. However, only a small number of studies have investigated the relationship between molecular classification and the efficacy of FST in young EC/AH patients, and the results are contradictory [7,8,9,10]. Therefore, there is an urgent need for further clinical research to elucidate the precise prognostic significance of molecular classification in these patients undergoing FST.

The most common molecular subtype of patients with EC and AH receiving FST is the NSMP subtype (64–86.7%), with the other three types having a prevalence of 10.4–19% for mismatch repair deficient (MMRd), 0.7–13% for polymerase ε-mutant (*POLE*mut), and 2.2–4% for p53mutant (p53abn) [11]. According to the ESGO/ESHRE/ESGE guidelines, patients with NSMP subtypes are suitable for FST [12]. For patients with the *POLE*mut subtype, conservative treatment remains unclear. Fertility preservation may also not be suitable for patients with p53abn due to the aggressive characteristic of the malignancy. Patients with MMRd are usually less responsive to progesterone therapy and have a high risk of recurrence after initial regression [12]. Since most patients with EC treated with FST are the NSMP subtype, there are very limited data on the outcome of FST in patients with the other three subtypes [13].

The aim of this single-center, retrospective study was to evaluate the prognostic value of molecular classification for EC/AH patients receiving FST. We also studied the treatment regimens and outcomes for patients with molecular subtypes other than NSMP to enhance the prognostic and therapeutic options in this special patient group.

## 2. Materials and Methods

### 2.1. Study Population 

This was a retrospective cohort study. Data on patients with stage Ia G1–G2 EC or AH who received FST and had molecular typing conducted by next-generation sequencing at Peking University People’s Hospital from June 2020 and September 2023 were collected. The inclusion criteria used were in accordance with the Chinese expert consensus and were as follows: (1) AH or well-differentiated adenocarcinoma grade 1–2 (stage IA disease, as determined by the 2009 FIGO), with positive estrogen receptor (ER) and progesterone receptor (PR) by immunohistochemical results; (2) EC without myometrial invasion (MI) or with superficial MI determined by magnetic resonance imaging (MRI); (3) no suspicion of lymph node metastases, ovarian tumor, or other metastasis; and (4) strong desire and consent for fertility-sparing treatment [14].

### 2.2. Fertility-Sparing Treatment

Patients were administered with hysteroscopic resection followed by progestin-based treatment, either 250–500 mg per day of continuous oral medroxyprogesterone acetate (MPA), 160–320 mg per day of megestrol acetate (MA), or continuous intrauterine placement of a levonorgestrel-releasing system (LNG-IUS) plus 3.75 mg of gonadotropin-releasing hormone agonist (GnRHa), injected subcutaneously once every 28 days. If a complete response (CR) was not obtained after 6–9 months, multidisciplinary discussions were carried out, and combined regimens were administered according to the recommendations of the doctors. For patients with MMRd with a strong will for fertility preservation, an initial progestin regimen was given before October 2022, and following that date, the patients were administered a combined regimen of progestin plus programmed death protein-1 inhibitors (PD-1i). PD-1i was administrated as 200 mg sindilizumab by intravenous drip, once every three weeks for a total of six cycles.

### 2.3. Follow-Up and Evaluation of Treatment Efficacy 

An endometrial specimen was obtained by hysteroscopic biopsy every three months during treatment. The pathological diagnosis was reviewed by 2 independent pathologists based on the 5th edition of the WHO Classification of Female Genital Tumors [15]. Treatment efficacy was evaluated by expert consensus [14]. CR was defined as the absence of hyperplasia or carcinoma, a partial response (PR) as pathological improvement and the presence of a hormone effect, and no response (NR) as the persistence of the originally diagnosed lesion. Progression of disease (PD) was defined as disease progression to a higher grade or progressive disease. Recurrence was defined as the reappearance of EC or AH after CR.

### 2.4. Molecular Classification Procedure

Paraffin-embedded tissue sections with lesions comprising more than 30% of the area were selected, and 5 μm thick slices were taken to extract the DNA. The polymerase chain reaction (PCR) was used to construct the sequencing library. Sequencing was carried out using the NGS Panel (Amoy Diagnostics, Xiamen, China), encompassing the *POLE* gene, TP53 gene, and 55 microsatellite loci by the NextSeq500 Illumina platform (Miseq, illumina, USA). This comprehensive panel enables the accurate detection of single-nucleotide variants, insertions, and deletions, as well as microsatellite instability (MSI) status. The simplified NGS Panel has been validated as a user-friendly and widely applicable tool, demonstrating high accuracy in EC molecular classification [16]. Then, the data were analyzed by a system purchased from Xiamen AmoyDx Biopharmaceutical Technology Co., Ltd. (Xiamen, China). The patients were divided into four subgroups using the NGS classification panel, namely *POLE*mut, MMRd, NSMP, and p53abn. The classification process was as follows: (1) The mutation status of the *POLE* gene was detected, and if it had mutated, the patient was then classified in the *POLE*mut subgroup. And the pathogenic mutation of the *POLE* gene (including 11 mutation sites: P286R, V411L, S297F, S459F, A456P, F367S, L424I, M295R, P436R, M444K, and D368Y) was identified as a *POLE* mutant subtype [17]. (2) The wildtype *POLE* gene was tested for MSI, and if more than 15% of the microsatellite loci exhibited instability, the patient was classified in the MSI-H/MMRd subgroup; otherwise, there was microsatellite stability. (3) The mutation status of the TP53 gene for patients with microsatellite stability was detected, and if it had mutated, the patient was classified in the p53abn subgroup, while those with no mutation were classified in the NSMP subgroup. 

### 2.5. Post-Treatment Management

After CR, the patients were encouraged to conceive with or without active assisted reproduction technology (ART) or offered maintenance treatment. After the completion of treatment, follow-up involving ultrasound or hysteroscopy if necessary was carried out every 3–6 months during the first 3 years and prolonged to 6 months in the following 2 years. The patients were followed up until December 2023. 

### 2.6. Statistical Analysis

Statistical analyses were performed using SPSS 25.0 software. Differences between two groups were compared using Student’s *t*-test or the Mann–Whitney U test. Differences between more than two groups were detected using one-way analysis of variance (ANOVA) or the Kruskal–Wallis *H* test where appropriate. The frequency distributions were compared using the Chi-squared test or Fisher’s exact test. The cumulative CR rate and relapse-free survival (RFS) rate were estimated by the Kaplan–Meier method, with intergroup differences compared by the log-rank test. A *p* value < 0.05 was regarded as statistically significant. 

## 3. Results

A total of 118 eligible EC/AH patients were investigated retrospectively. The NSMP subgroup was the main molecular classification with a total of 92 cases (78%), while 11 cases (9.3%) had the MMRd subtype, 11 cases (9.3%) had the POLEmut subtype, and 4 cases (3.4%) had the p53abn subtype.

Of the 118 patients, 81 patients (68.6%) achieved CR with a median treatment duration to CR of 7 months (range, 3.5–12 months). The median follow-up time from the date of achieving CR to the last follow-up was 9 months (range, 4–18.5 months). Twenty of the eighty-one patients (16.9%) with CR relapsed, with a median time to recurrence of 9.5 months (range, 7–18.3 months). 

The characteristics of the patients are presented in Table 1. Patients with the NSMP subtype tended to be younger among the four subtypes and had a higher body mass index (BMI). However, these differences were not significantly different. Specifically, the patients in the NSMP group had the lowest serum levels of HDL-C (*p* = 0.010). The MMRd subtype had a slightly higher family history of tumors than that in the other three groups (*p* = 0.068). For treatment regimens, notably, more patients with MMRd received immune checkpoint inhibitor (ICI) therapy (45.5%). Regarding treatment outcomes, there were no significant differences in CR rates and the time to achieve CR between the four subgroups. However, disease progression occurred more frequently in the MMRd group (27.3%), while patients with POLEmut tended to have a higher CR rate (90.9%) and also a higher recurrence rate (40%).

### 3.1. Outcomes for Patients with MMRd 

The therapeutic effects for the 11 patients with MMRd are shown in Table 2. Four patients underwent a hysterectomy, including one (case 1) with PD, one with SD (case 2), and two recurrent cases (cases 3 and 4). One patient showed PD during treatment but refused radical surgery and was alive after one year of follow-up (case 5).

Another six cases achieved CR after FST. While case 6 with AH achieved CR after MPA treatment, another three patients (cases 7–9) were all insensitive to progestin and were given the combined regimen of progestin plus PD-1i, which resulted in all achieving CR. Cases 10 and 11 initially received the combined regimen and achieved CR after 3 and 7 months of treatment, respectively. Temporarily, none of these six patients attempted to conceive after a median follow-up time of 5 months since CR.

**Figure 1:**
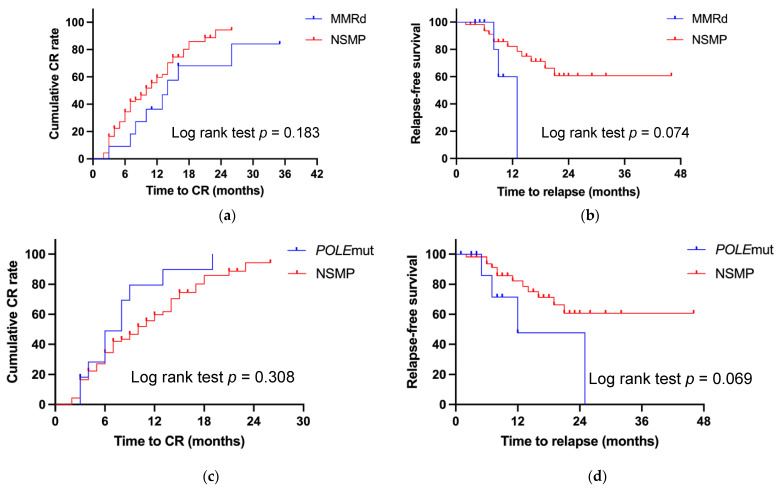
Kaplan–Meier analysis compared by the log-rank test of the cumulative CR rate and relapse-free survival. (**a**) Treatment time to CR and (**b**) relapse-free survival between the MMRd and NSMP molecular classifications. (**c**) Treatment time to CR and (**d**) relapse-free survival between POLEmut and NSMP molecular classifications.

The Kaplan–Meier analysis is shown in Figure 1. And Figure 1a indicates lower cumulative CR rates for patients with MMRd, compared with those with NSMP, but with no statistical difference (*p* = 0.183) (Figure 1a), which may have been due to the change in treatment regimen from progestin to ICIs. There was a trend of a lower relapse-free survival (RFS) rate for the MMRd subtype compared with NSMP (*p* = 0.074) (Figure 1b).

### 3.2. Outcomes for Patients with POLEmut

A total of 11 POLEmut patients were enrolled in this study (Table 3). Ten patients achieved CR, with the median time to CR being seven months. 

Four of the ten CR patients relapsed after 5, 6, 12, and 25 months from CR, with three receiving a second round of treatment to achieve CR again and one receiving staging surgery (cases 12, 15, 19, and 22).

Two cases developed ovarian cancer during FST (cases 16 and 22), with postoperative pathology confirming the presence of ovarian EC. 

Kaplan–Meier analysis showed no significant difference in the cumulative CR rates for patients with POLEmut and NSMP (*p* = 0.308) (Figure 1c). There also appeared to be a trend for a lower RFS rate in the POLEmut patients, although this difference was not statistically significant (*p* = 0.069) (Figure 1d).

### 3.3. Outcomes for Patients with p53abn

A total of four patients with p53abn were included in this study (Table 4), all of whom gave fully informed consent before FST. 

Three patients (cases 23–25) were treated with GnRHa + LNG-IUS, with all achieving CR. However, case 23 was given IVF-ET and achieved a live birth, although an ovarian seromucinous borderline tumor was incidentally found during a cesarean section. Case 24 showed focal hyperplasia of the endometrium during follow-up. Case 26 had stable disease after the initial treatment of GnRHa + LNG-IUS and was then given chemotherapy and is still undergoing treatment. 

The oncologic outcomes could not be compared because of the small number of patients in this group.

## 4. Discussion

This study evaluated the guiding value of molecular classification for EC/AH patients undergoing FST. Our study consisted of the greatest number of patients with subtypes other than NSMP and also described their detailed treatment outcomes. Furthermore, this study provided preliminary proof of the effectiveness of treatment with a PD-1 inhibitor in EC/AH patients with the MMRd subtype receiving FST.

The current study showed that molecular classification was associated with the clinicopathological characteristics of patients. In accordance with Britton and Raffone’s studies [11,18], we showed that patients with the p53wt subtype were younger and had the highest BMI. A similar trend was observed in these subgroups within Western populations [7].

Overweight and obesity were identified as independent risk factors that affect the duration of treatment. Also, the patients in the NSMP group had the lowest serum levels of HDL-C (*p* = 0.010). Therefore, we consider that NSMP EC/AH tumors may be related to risk factors for metabolic abnormalities and are more “high estrogen like”. As treatment response differs among the NSMP subgroup, the refinement of NSMP EC/AEH using estrogen receptor immunohistochemistry, grade, and CTNNB1 mutation might add more information for guiding FST in such patients [19,20,21].

In contrast to most literature showing poor treatment results for MMRd patients, we proved the promise of a regimen of ICIs in such groups. Wang’s study [22] demonstrated that MMRd patients all failed to achieve CR within 6 months (7/7), with a significantly lower CR rate (*p* = 0.014). Similarly, Zakhour’s study [23] showed that of the 84 patients with EC/AH who received FST, only 6 (7%) were MMRd patients and that the CR rate was significantly lower than that of non-MMRd patients (0 vs. 53%; *p* = 0.028) [23]. In addition, a recent study that included 11 MMRd patients with AH showed that patients with MMR-d had lower 12-month CR rates, higher relapse rates at 1-year follow-up, and a higher incidence of disease progression than patients with normal staining patterns [24]. However, Ran and Xu’s studies [10,13] showed there was no significant difference in the complete response rate and recurrence rate between MMRd and p53wt subtypes after FST. In our study, Kaplan–Meier survival curve analysis revealed no significant difference in cumulative CR rates between the MMRd and NSMP subgroups, with this finding possibly related to a change in the treatment regimen to ICI therapy. Notably, we found that three cases were insensitive to initial progestin treatment and that all achieved CR after combined therapy with ICIs, whereas another two cases achieved CR with the initial ICI therapy. Evidence from other studies shows that the MMRd group had a higher recurrence rate, 50.0–100%, than MMR-proficient cases [9,10,13]. In our cohort, we showed slightly higher recurrence rates (25% vs. 21.7%) and lower RFS rates (*p* = 0.074) for the MMRd group compared with the NSMP groups. It is therefore important in the future to further prove the oncological prognosis of fertility preservation within the MMRd group, and especially to prove the efficacy of ICI therapy.

We found patients with *POLE*mut have a high recurrence risk (40%) and a lower RFS rate after FST, unlike their favorable postoperative prognosis. Xu’s study [13] also showed that patients with *POLE*mut had the highest disease progression rate of 50.0% (*p* = 0.013). Also, in Puechl’s study [25], 1 of 4 (25%) of AH patients with *POLE*mut and 2 of 27 (7.4%) patients with p53wt demonstrated disease progression. We also showed that two cases with *POLE*mut experienced ovarian cancer during treatment, which made their fertility preservation more challenging. These data suggested that the *POLE* mutation may be one of the unfavorable factors for fertility preservation in EC/AH patients. However, further studies are essential to investigate the best treatment regimen and follow-up strategies.

Patients with the p53mut subgroup have the worst prognosis, with a high risk of recurrence and total survival, and therefore, these patients are not supposed to be treated conservatively [26]. However, four patients in the p53mut subgroup in this study received FST using a regimen of GnRHa plus LNG-IUS. Three patients achieved CR, with one becoming pregnant. However, during the follow-up period, we found that ovarian or endometrial lesions had occurred in two patients. Some patients with the p53abn subgroup may achieve remission following hysteroscopic lesion resection, which minimizes tumor burden. However, it has been reported that AH lesions may progress to EC after management with LNG-IUS [27]. Other studies [13,24] showed a poor treatment response and a significantly higher risk of disease relapse and progression for patients with p53abn. However, evidence on the safety of fertility-preserving treatment or the best therapy is rather limited in this group of patients.

This study had some merits. First, we included a relatively large patient cohort and showed the relationship of clinicopathological characteristics with molecular classification and its guiding value for individualized treatment for fertility-preserving patients. Second, this study consisted of the greatest number of patients other than the NSMP subtype and described their detailed treatment outcomes. We consider that this study will enhance the prognostic and therapeutic options in this special patient group. In particular, we were the first to prove the effectiveness of treatment with a PD-1 inhibitor in MMRd patients for fertility preservation. 

However, this study does have several limitations. First, it had a retrospective design and used a single institution database including eight AEH patients. Although reported in some of the literature, the evidence of the application of molecular classification in AH patients is still limited [28]. Second, the patients received different regimens rather than standard treatment. This was because some patients had complicated metabolic status or were insensitive to progestin. In these patients, we tried combined therapies such as GnRHa, metformin, or ICI regimens. Third, the small sample size of the three subgroups and also the specific Chinese population may have restricted the generalizability of our findings, although our preliminary results may be used to improve further investigations.

## 5. Conclusions

This study used the TCGA molecular classification to confirm associations of molecular subtypes with phenotypes and treatment efficacy for EC or AH patients receiving FST. We demonstrated that patients with MMRd were resistant to progestin treatment but obtained significant benefits from ICI therapy. For patients with *POLE*mut, although their CR rate was high, disease relapse occurred more frequently. For the smallest group, with the p53mut subtype, patients may achieve remission from individuated treatment regimens. Steps towards individual therapy for these non-NSMP EC/AH patients are worthwhile and encouraging in order to improve the outcomes of fertility preservation.

## Figures and Tables

**Table 1:** Clinicopathological characteristics of the patients in the four molecular subgroups.

Variable	*POLE*mut *n* = 11	MMRd *n* = 11	NSMP *n* = 92	p53abn *n* = 4	*p* Value
Pathology, *n* (%)					0.867
AH	1 (9.1)	1 (9.1)	6 (6.5)	0	
EC G1	7 (63.6)	9 (81.8)	73 (79.3)	3 (75)	
EC G2	3 (27.3)	1 (9.1)	13 (14.1)	1 (25)	
Age (years)	37 (30–42)	37 (30–39)	33 (29–37)	34 (25.5–44)	0.184
BMI (kg/m^2^)	23.6 (21.1–26.6)	21.4 (20.4–26.1)	25.9 (22.1–31.1)	27.6 (22–28)	0.089
Pregnancy history, *n* (%)	4 (36.4)	3 (27.3)	23 (25)	1 (25)	0.890
Parity, *n*(%)	1 (9.1)	1 (9.1)	12 (13)	1 (25)	0.882
Waist (cm)	84 (72–93)	82.5(71.8–102.5)	84 (78–97)	87.5 (71–93.5)	0.703
Hip (cm)	96 (89–107)	96.5 (90.8–112.8)	100.5 (94.3–111.4)	100 (94–102.3)	0.569
Diabetes, *n* (%)	1 (9.1)	2 (18.2)	11 (12)	1 (25)	0.826
IR *n* (%)	3 (27.3)	4 (40)	45 (51.1)	1 (25)	0.331
Hypertension, *n* (%)	1 (9.1)	0	12 (13)	0	0.328
Hyperlipidemia, *n* (%)	4 (50)	5 (71.4)	45 (57)	1 (25)	0.487
HDL-C (mmol/L)	1.3 (1–1.6)	1.3 (1.2–1.5)	1.1 (1–1.3)	1.4 (1.3–1.7)	0.010
LDL-C (mmol/L)	2.7 (2.2–3.1)	3.5 (2.6–4.1)	2.8 (2.4–3.4)	2.7 (2.2–3.5)	0.185
Triacylglycerol (mmol/L)	1 (0.9–1.1)	1.5 (0.9–3.7)	1 (0.8–1.5)	0.8 (0.7–1.1)	0.224
Cholesterol (mmol/L)	4.3 (4.2–4.7)	4.6 (3.5–5.7)	4.6 (4.1–5.2)	4.5 (4.1–5.9)	0.987
PCOS, *n* (%)	2 (18.2)	1 (9.1)	21 (23.9)	1 (25)	0.658
Thyroid disease, *n* (%)	1 (9.1)	0	9 (9.8)	0	0.414
Family history of cancer, *n* (%)	4 (36.4)	6 (54.5)	22 (23.9)	0	0.068
CA-125 (U/mL)	11 (6.9–17.8)	18.1 (9.8–26.8)	15.4 (10.3–22.4)	17 (7.6–50.6)	0.353
Therapy					0.000
MPA/MA	46 (60)	64 (36.4)	39 (54.3)	1 (25)	
MPA/MA→GnRHa + LNG-IUS	2 (20)	2 (18.2)	36 (40.9)	0	
GnRHa + LNG-IUS/letrozol	1 (10)	0	13 (14.8)	3 (75)	
Combined with ICI	1 (10)	5 (45.5)	0	0	
Therapy outcomes, *n* (%)					
CR	10 (90.9)	8 (72.7)	60 (65.2)	3 (75)	0.284
PD	0	3 (27.3)	1 (1)	0	0.007
Recurrence	4 (40)	2 (25)	13 (21.7)	1 (33.3)	0.671
Time to CR (months)	7 (3.8–10)	11 (7.3–15.5)	6.5 (3.3–12)	9 (3–12)	0.383
Follow-up period (months)	8.5 (3.8–13.8)	5 (0–9)	10.5 (4–21)	6 (1–11)	0.367

AH: atypical endometrial hyperplasia, EC: endometrial cancer, BMI: body mass index, IR: insulin resistance, HDL-C: high-density lipoprotein cholesterol, LDL-C: low-density lipoprotein cholesterol, PCOS: polycystic ovary syndrome, MPA: medroxyprogesterone acetate, MA: megestrol acetate, GnRHa: gonadotropin-releasing hormone agonist, LNG-IUS: levonorgestrel-releasing system, ICI: immune checkpoint inhibitor, CR: complete response, PD: progression of disease.

**Table 2:** General information and treatment outcomes for the 11 patients with the MMRd subtype.

Case No.	Diagnosis	BMI kg/m^2^	Complications	Deficient MMR Protein	Regimen	Time to CR (Months)	Oncological Outcomes
1	ECG2	21.3	LS	MLH1/ PMS2-	MPA 250 → 500 mg, 6 m, SD MPA 500 mg, 6 m, PR GnRHa + LNG-IUS, 12 m, CR → SD GnRHa + LNG-IUS, 6 m, SD Chemo TC × 2, AP × 1, 3 m, PD	/	PD Staging surgery Endometrial dedifferentiated cancer IIIC1
2	ECG1	28.2	LS	MSH2/ MSH6-	MA 320 mg, 3 m, SD MPA 500 mg + GnRHa + metformin, 9 m, SD MPA 500 + GnRHa + metformin, 2 m	/	NR Staging surgery EC G1 Ia Ovary endometrioid cancer G1 Ia
3	ECG1	22.2	Diabetes LS	MLH1/ PMS2-	LNG-IUS, 3 m, SD MA160 mg + LNG-IUS + metformin, 5 m, SD GnRHa + letrozole, 6 m, CR Recurrence 8 m after CR	13	Recurrence Staging surgery Pathology unknown
4	ECG2	22.3	Breast cancerLS	MSH2/ MSH6-	MPA250 mg, 3 m, SD MPA250 mg + GnRHa, 9 m, CR Recurrence 13 m after CR, ECG1	12	Recurrence Staging surgery EC G2 Ia
5	ECG2	21.4	None	MSH2/ MSH6-	MPA500 mg, 1 m, MPA500 mg + chemo TC × 2, 2 m, PD MPA500 mg + IAP×6 + PD-1, 6 m, SD Refused following treatment	/	PD Survival at 12-month follow-up
6	AH	18.4	LS	MSH2/ MSH6-	MPA 500 mg, 5 m, SD MPA 500 mg, 3 m, CR	8	CR
7	ECG2	34.5	DM, PCOS	PMS2-	MPA 500 mg + metformin, 3 m, PR Chemo AP × 2 + MPA 500 mg + metformin, 4 m, PR GnRHa + LNG-IUS, 6 m, PR GnRHa + LNG-IUS + letrozole + PD-1i + metformin, 6 m, PR GnRHa + LNG-IUS + letrozole + PD-1i + metformin + statin, 6 m, CR	25	CR
8	ECG1	26.0	None	MSH6-	MA 320 mg, 4 m, PR MPA 500 mg + GnRHa + LNG-IUS, 2 m, MPA 500 mg + GnRHa + LNG-IUS + PD-1i, 2 m CR, 3 m CR	8	CR
9	ECG2	19.83	None	MSH2/ MSH6-	MPA 500 mg, 11 m, PR GnRHa + LNG-IUS + PD-1i, 3 m, CR	14	CR
10	ECG1	16.6	None	MSH2/ MSH6-	MPA 250 mg + PD-1i, 3 m, CR	3	CR
11	ECG1	20.4	None	MSH6-	MPA 250 mg + GnRH + PD-1i, 4 m, PR GnRH + LNG-IUS + PD-1i, 3 m, CR	7	CR

AH: atypical endometrial hyperplasia, EC: endometrial cancer, BMI: body mass index, LS: Lynch Syndrome, PCOS: polycystic ovary syndrome, MPA: medroxyprogesterone acetate, MA: megestrol acetate, GnRHa: gonadotropin-releasing hormone agonist, LNG-IUS: levonorgestrel-releasing system, chemo: chemotherapy, TC: paclitaxel/carboplatin, AP: doxorubicin/cisplatin, IAP: ifosfamide/doxorubicin/cisplatin, PD-1i: programmed death protein-1 inhibitor, CR: complete response, PR: partial response, NR: no response, PD: progression of disease.

**Table 3:** General information and treatment outcomes for the 11 patients with the *POLE*mut subtype.

Case No.	Diagnosis	MI	BMI kg/m^2^	*POLE* Mutation Sites	Regimen	Time to CR (Months)	Oncological Outcomes
12	ECG2	Yes, Superficial, Intraperitoneal metastasis	19.7	S459F	MPA 500 mg + chemo, 6 m CR AH recurrence 25 m after CR	6	CR IVF-ET Live birth Recurrence and CR
13	AH	No	25.5	P286R	MPA 250 → 500 mg + metformin, 14 m CR	14	CR IVF-ET ongoing
14	ECG1	No	28.6	P286R	GnRHa + LNG-IUS, 3 m PR GnRHa + LNG-IUS + MPA 250 mg, 5 m CR	8	CR No fertility plan
15	ECG1	No	32.1	V411L	MPA 250 mg + metformin 4 m CR Recurrence after 6 m MPA 250 mg + metformin + GnRHa + LNG-IUS, 10 m CR	4	CR Recurrence and CR IVF-ET ongoing
16	ECG1	No	26.6	P286R	MPA 500 mg + metformin, 5 m, CR Ovarian tumor 5 m after CR, chemo TC × 4	6	CR Ovary endometrioid cancer G1 Ic1 IVF-ET ongoing
17	ECG1	No	23.6	P286R	MPA 250 mg 3 m CR	3	CR
18	ECG2	Yes, Superficial	26.0	L424I	MPA 500 mg + chemo TC + GnRHa + PD-1i, 3 m CR	3	CR
19	ECG1	No	23.0	Unknown	MA 160 → 320 mg + LNG-IUS, 13 m SD, GnRHa + letrozole, 6 m CR AH recurrence 5 m after CR GnRHa + letrozole + LNG-IUS, 3 m CR	19	CR Recurrence and CR Pregnant, 11-week gestation
20	ECG2	Yes, Superficial	18.9	V411L	MPA 500 mg + LNG-IUS 3 m, PR GnRHa + LNG-IUS + metformin	/	In treatment
21	ECG1	No	21.5		MPA 250 mg, 6 m, CR	6	CR IVF-ET ongoing
22	ECG1	No	21.1	V411L	MPA 250 mg, 3 m, PR MPA 250 mg + metformin, 3 m, PR+ Ovarian endometrioid adenocarcinoma G1 MPA 250 mg + metformin, 9 m, CR ECG1 recurrence 12 m after CR	9	Ovary endometrioid cancer G1 Ia CR Recurrence and staging surgery

AH: atypical endometrial hyperplasia, EC: endometrial cancer, BMI: body mass index, MI: myometrial invasion, MPA: medroxyprogesterone acetate, GnRHa: gonadotropin-releasing hormone agonist, LNG-IUS: levonorgestrel-releasing system, chemo: chemotherapy, PD-1i: programmed death protein-1 inhibitor, CR: complete response, PR: partial response, IVF-ET: in vitro fertilization–embryo transfer, TC: paclitaxel/carboplatin.

**Table 4:** General information and treatment outcomes for the four patients with the p53mut subtype.

Case No.	Diagnosis	MI	BMI	Regimen	Time to CR (Months)	Oncological Outcomes
23	ECG2	<1/2 MI	20.2	GnRHa + LNG-IUS, 3 m CR	3	CR IVF-ET Cesarean section Ovarian borderline tumor
24	ECG1	None	27.5	MPA250 9 m PR GnRHa + LNG-IUS, 3m CR	12	CR Focal hyperplasia 6 m after CR
25	ECG2	None	28.4	GnRHa + LNG-IUS, 3 m PR GnRHa + LNG-IUS + MPA 250 mg, 6 m CR	9	CR
26	ECG1	None		GnRHa + LNG-IUS, 3 m NR Chemo TC × 4, PR	/	Treatment

EC: endometrial cancer, MI: myometrial invasion, BMI: body mass index, MPA: medroxyprogesterone acetate, GnRHa: gonadotropin-releasing hormone agonist, LNG-IUS: levonorgestrel-releasing system, CR: complete response, PR: partial response, NR: no response, IVF-ET: in vitro fertilization–embryo transfer, chemo: chemotherapy, TC: paclitaxel/carboplatin.

## Data Availability

The data presented in this study are available on request from the corresponding author.
